# Superior Cervical Ganglia Neurons Induce Foxp3+ Regulatory T Cells via Calcitonin Gene-Related Peptide

**DOI:** 10.1371/journal.pone.0152443

**Published:** 2016-03-29

**Authors:** Kirsten Szklany, Evelyn Ruiter, Firoz Mian, Wolfgang Kunze, John Bienenstock, Paul Forsythe, Khalil Karimi

**Affiliations:** 1 The Brain Body Institute, McMaster University, Hamilton, Ontario, Canada; 2 Department of Medicine, McMaster University, Hamilton, Ontario, Canada; 3 Department of Pathology and Molecular Medicine, McMaster University, Hamilton, Ontario, Canada; Wayne State University, UNITED STATES

## Abstract

The nervous and immune systems communicate bidirectionally, utilizing diverse molecular signals including cytokines and neurotransmitters to provide an integrated response to changes in the body’s internal and external environment. Although, neuro-immune interactions are becoming better understood under inflammatory circumstances and it has been evidenced that interaction between neurons and T cells results in the conversion of encephalitogenic T cells to T regulatory cells, relatively little is known about the communication between neurons and naïve T cells. Here, we demonstrate that following co-culture of naïve CD4^+^ T cells with superior cervical ganglion neurons, the percentage of Foxp3 expressing CD4^+^CD25^+^ cells significantly increased. This was mediated in part by immune-regulatory cytokines TGF-β and IL-10, as well as the neuropeptide calcitonin gene-related peptide while vasoactive intestinal peptide was shown to play no role in generation of T regulatory cells. Additionally, T cells co-cultured with neurons showed a decrease in the levels of pro-inflammatory cytokine IFN-γ released upon *in vitro* stimulation. These findings suggest that the generation of Tregs may be promoted by naïve CD4^+^ T cell: neuron interaction through the release of neuropeptide CGRP.

## Introduction

The nervous and immune systems, communicate through the production of signaling molecules such as cytokines and neurotransmitters [1, 2]. Neurons release neurotransmitters, the receptors for which are expressed by cells of both the innate and adaptive immune systems [1, 3], and immune cells influence the nervous system by the release of cytokines that directly or indirectly communicate with the nervous system [4–6]. Neurons have been shown to regulate T cell function [7, 8] and neuron-T cell interaction can increase survival of neurons [2].

T cells largely regulate adaptive immune responses [9]. CD4^+^ T cells can be subdivided from a functional point of view into two main subsets. Effector cells provide protection against exogenous offending agents, and regulatory T (Treg) cells whose function is to avoid autoimmune reactions and to stop effector responses against exogenous antigens, when the response itself becomes dangerous for the host. Effector CD4^+^ T cells include T helper (Th) 1, Th2, Th17, and Th22 [10, 11] and the differentiation of naive T cells into the different subsets is regulated by the presence of environmental cytokines; for instance, interleukin 12 (IL12) and interferon γ (IFNγ) are the critical cytokines initiating the downstream signaling cascade to develop Th1 cells, while Treg differentiation is promoted by TGF-β in the absence of IL-6 [11–14].

Tregs play an important role in regulating immune homeostasis and tolerogenesis, as well as preventing autoimmunity [15]. Their dysfunction can lead to a number of immunopathologies such as allergies and autoimmune diseases including type-1-diabetes and multiple sclerosis [15]. Tregs are characterized by expression of the transcription factor forkhead box p3 (Foxp3), and the surface marker CD25 that is the IL-2 receptor α-chain [16]. Tregs are known to regulate a number of cellular components and activity in both innate and adaptive immune responses. These CD4^+^CD25^+^Foxp3^+^ Tregs can be further classified into different subtypes; natural Tregs (nTregs) and induced Tregs (iTregs). nTregs are derived from the thymus and iTregs are differentiated from naïve T cells after antigen stimulation in presence of TGF-β in the periphery [17]. Both of these two types of regulatory T cells maintain immune tolerance and prevent the occurrence of inflammatory diseases [15, 18]. It has been widely assumed that the generation of Tregs occurs exclusively within the immune system however neurons and other cells in the nervous system are capable of synthesis of cytokines such as IL-6 [19] and receptors for molecules such as IL-10 [20]. Indeed when neurons are co-cultured with encephalitogenic T cells, the production of TGF-β by neurons induces Foxp3^+^ T regulatory cells with the capacity to suppress autoreactive T cells [2]. However, the potential of the nervous system to influence normal non-neuroreactive T cells is not known. We have co-cultured normal superior cervical ganglia (SCG) with naïve T cells and investigated the induction of T regs by neurons in this co-culture system. We found that interaction between neurons and T cells results in Foxp3 expression in the T cells, accompanied by down-regulation of IFNγ expression in CD4^+^T cells. Moreover, we found that the induction of Foxp3 expression in T cells is mediated by the neurotransmitter calcitonin gene-related peptide (CGRP) as well as the regulatory cytokines TGF-β and IL-10.

## Methods

Animals: 14–16 days pregnant BALB/c mice were purchased from Charles River Laboratories (Quebec, Canada). The mice were housed at 25°C on a 12hr light/dark cycle in individual vented caging (IVCs), 1 pregnant mouse per cage in a specified pathogen free room, with food and water ad libitum. All animal care and experimental procedures were performed according to the requirements of the Animal Care Committee of McMaster University and approved by McMaster's Animal Research Ethics Board (AREB) AUP #13-05-15, Hamilton, Ontario, Canada. Animals were monitored for health every day before use. Carbon dioxide (CO_2_) inhalation was used for euthanasia of mice. Neonates (1–3 days after birth) were used for the isolation of SCG [21].

SCG culture: The ganglia were placed in Puck’s saline after isolation, and transferred to calcium and magnesium free Puck’s saline containing 0.25% trypsin (Gibco) and incubated for 30 min at 37°C. The ganglia were transferred to 0.2% collagenase Type 1A (Sigma), incubated for another 10 minutes and washed twice with Puck’s saline containing 10% FBS (Gibco, USA). The ganglia were dissociated by trituration using a 5 ml serological pipet (Falcon) attached to a 1 ml Eppendorf pipet tip. After dissociation the cells were centrifuged and resuspended in DME/F12 medium (Gibco) containing 10% FBS (Gibco origin USA), 0.5% penicillin/streptomycin (Gibco) and 1% glutamine (Gibco). 100 ng/ml 2.5 S nerve growth factor (NGF) (Cedarlane) and 1 uM cytosine arabinoside (Sigma) were added to the cell suspension to sustain neuron cell growth and inhibit non-neuronal cell growth, respectively. The neuron cells were grown in rat tail collagen- (BD Biosciences) and poly-L-ornithine- (Sigma) coated 24 well plates (1 ganglion per well) or 96 well plates (1 ganglia per 3 wells) (Falcon).

Co-culture: Total splenocytes, purified CD4^+^ T cells, or CD4^+^CD62L^+^ T cells were added to the neuron culture on day 2. Spleens were mashed through a 40μm filter (Falcon) and a single-cell suspension was made before lysing the red blood cells using a lysis buffer (eBioscience). 0.5x10^6^ total splenocytes were added to the neuron culture in presence and absence of recombinant interleukin 2 (IL-2) (R&D systems) at a concentration of 200 U/ml. When purified T cells were used, the splenocytes were purified using CD4^+^ or CD4^+^CD62L^+^ cell isolation kits (Miltenyi biotec) and no lysis buffer was used. 0.3x10^6^ purified cells were added to the neuron culture on day 2 including 200 U/ml IL-2. Splenocytes, purified CD4^+^ T cells, or CD4^+^CD62L^+^ T cells alone were cultured as controls. One hour before addition of T cells to the neuronal cultures, the neutralization antibodies against NGF (8μg/ml, R&D Systems), TGF-β (1μg/ml, R&D systems), IL-10 (1μg/ml, BD Biosciences) or VIP (1x10^-6^ M, R&D Systems) or CGRP antagonist (1x10^-6^ M, Abcam) were incubated with the neurons. 0.5x10^6^ CD4^+^ or CD4^+^ CD25^-^ T cells were purified using Miltenyi biotec kits and incubated with different concentrations of CGRP (10^−14^ to 10^−8^) for 33 days in presence of 200 U/ml IL-2. T cell culture with IL-2 in absence or presence of CGRP antagonist (1x10^-6^ M, Abcam) used as controls.

Flow-cytometry: Foxp3 expressing Tregs were defined as CD3^+^CD4^+^CD25^+^Foxp3^+^ cells and IFNγ producing cells as CD3^+^CD4^+^IFNγ^+^ cells. Antibodies for CD3 (APC-Cy7), CD4 (FITC), CD25 (PE-Cy7) were used to stain the surface markers of the Foxp3 expressing Tregs (eBiosciences, USA). Cells were fixed and permeabilized (BD Biosciences and eBioscience) prior to intracellular staining with antibodies against Foxp3 (PE) (eBioscience, USA). Data were acquired with FACS Canto (BD Biosciences, Oakville, Canada) and analyzed with the FlowJo program (TreeStar, Ashland, OR, USA). For cytokine determination T cells were re-stimulated with 10 ng/ml Phorbol 12-myristate 13-acetate (PMA)(Sigma) and 1 μg/ml ionomycin (Sigma) for 2 hours at 37°C in 5% CO_2_ after which 1 ul/ml 0.1% Brefeldin A (Golgi-plug BD Biosciences) was added for an additional 4 hours under the same conditions. Subsequently the cells were stained with CD3 (APC-Cy7) and CD4 (APC) (eBioscience, USA), fixed and permeabilized and stained with antibody against IFNγ (PE-Cy7) (BD Biosciences). Cells were analyzed by flow cytometry, as mentioned above. The gating strategy was based on unstained controls and/or fluorescence-minus-one (FMO) controls [22]. Single and live events were gated based on forward scatter and side scatter plots and within lymphocyte region CD3+ T cells were gated out. Percentage of Foxp3^+^ cells was defined within CD3^+^CD4^+^ CD25^+^ T cells.

Statistical analysis: All statistical analyses were performed using Graphpad Prism 5 software. 1-way ANOVA followed by Bonferroni post-test was used to determine statistical differences between multiple groups. Unpaired Students t-test was used when only 2 groups were compared. All values are presented as mean ± SEM. A p-value <0.05 was considered significant.

## Results

### Co-cultured SCG-neurons increase the frequency of Foxp3^+^ T cells

**Fig 1:**
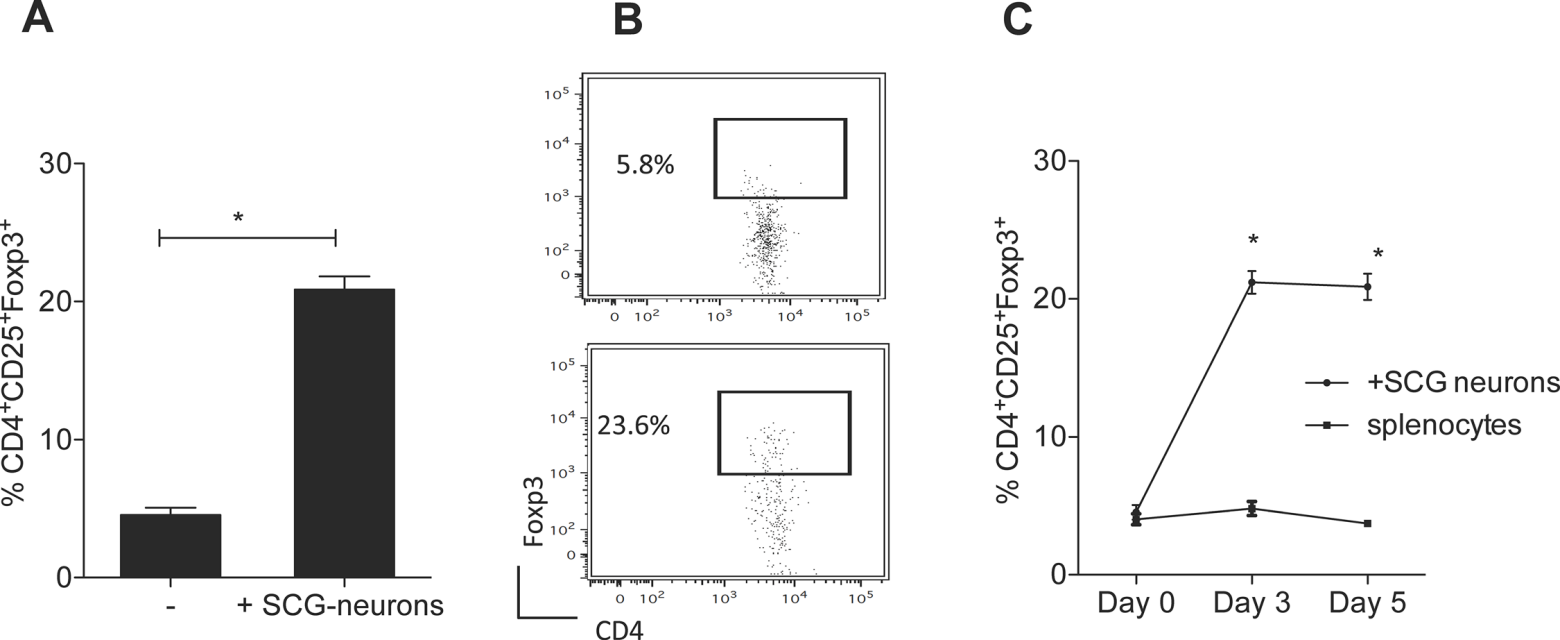
SCG-neurons increase the frequency of Foxp3^+^ T cells. Total splenocytes were co-cultured with SCG-neurons for 5 days and stained with antibodies against CD3, CD4, CD25, and Foxp3; and analyzed by flow cytometry. (A) Representative dot plots and (B) mean ± SEM percentage of Foxp3^+^ cells among the CD3^+^CD4^+^CD25^+^ cells are depicted. (C) Mean ± SEM percentage of Foxp3^+^ cells among the CD3^+^CD4^+^CD25^+^ cells in different time points in the co-culture is shown. (n = 4; *p<0.0001).

A five-day SCG neuron-splenocyte co-culture led to an increase in the percentage of Foxp3^+^ cells within CD4^+^CD25^+^ T cells determined by flow cytometric analysis. While the percentage of Foxp3^+^ cells in CD4^+^CD25^+^ T cells cultured in the absence of neurons was 4.54±0.52%, the percentage of cells that expressed Foxp3 within CD4^+^CD25^+^ T cells was increased to 20.88±0.94% (Fig 1A and 1B). The time-course co-culture experiments showed that the up-regulation of Foxp3 expression could be detected as early as the third day of co-culture (Fig 1C).

**Fig 2:**
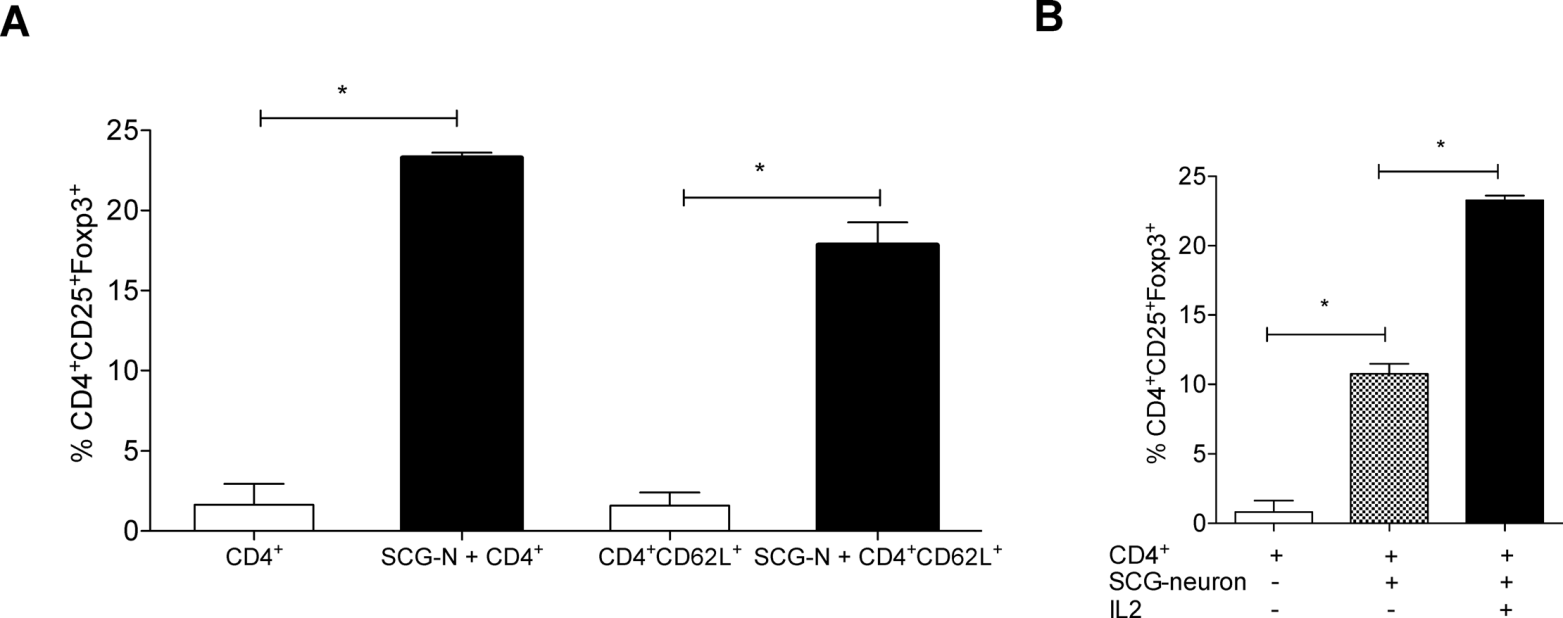
SCG-neurons generate Tregs from naïve T cells that is potentiated by IL-2. Purified CD4^+^ T cells or CD4^+^CD62L^+^ naïve T cells from total splenocytes from BALB/c mice were co-cultured with SCG-neurons and stained with antibodies against CD3, CD4, CD25 and Foxp3; and analyzed by flow cytometry. (A) Mean ± SEM percentage of Foxp3^+^ cells among the CD3^+^CD4^+^CD25^+^ cells are depicted. (CD4^+^ T cells n = 5, CD4^+^CD62L^+^ cells n = 5; * = p<0.0001). (B) Mean ± SEM percentage of Foxp3 expressions in CD3^+^CD4^+^CD25^+^ cells in the co-cultures in presence or absence of IL-2 are shown (n = 5, * = p<0.0001).

To further investigate neuronal generation of Tregs, purified CD4^+^ T cells were co-cultured with neurons and percentage of Foxp3 expression was detected by flow cytometry. Foxp3 expression significantly increased in CD4^+^ T cells in the co-cultures (23.27±0.33%) compared to purified CD4^+^ T cells that were left in culture media without neurons (1.637±1.30) (Fig 2A). No increase of Foxp3 expression was demonstrated in CD4^+^ T cells cultured with adherent cells isolated from spleen of syngeneic mice or the murine intestinal epithelial cell line MODE-K (S1 Fig). As CD4^+^ T cells may already contain Tregs, we sought to investigate whether the observed increase in Foxp3 expression after the co-culture could be due to the selective promotion of survival and proliferation of these existing Tregs. Therefore, purified naïve T cells, CD4^+^CD62L^+^ cells were co-cultured with neurons. Again, a significant increase in the percentage of Foxp3^+^ cells was detected in CD4^+^CD25^+^ T cells (17.83±1.41% p<0.0001) compared to CD4^+^CD62L^+^ T cells culture (1.576±0.83%) (Fig 2A). Interleukin-2 (IL-2) is critical for Treg development, expansion, activity and survival [23]. Addition of a low concentration of IL-2 (200U/ml) to the culture media showed a higher recovery of Foxp3 expressing cells (Fig 2B), in the neuron-T cell co-culture (10.75±0.73% to 23.27% ±0.33).

### Neuronal generation of Tregs is independent of nerve growth factor (NGF) and other soluble factors produced by neuronal culture alone

**Fig 3:**
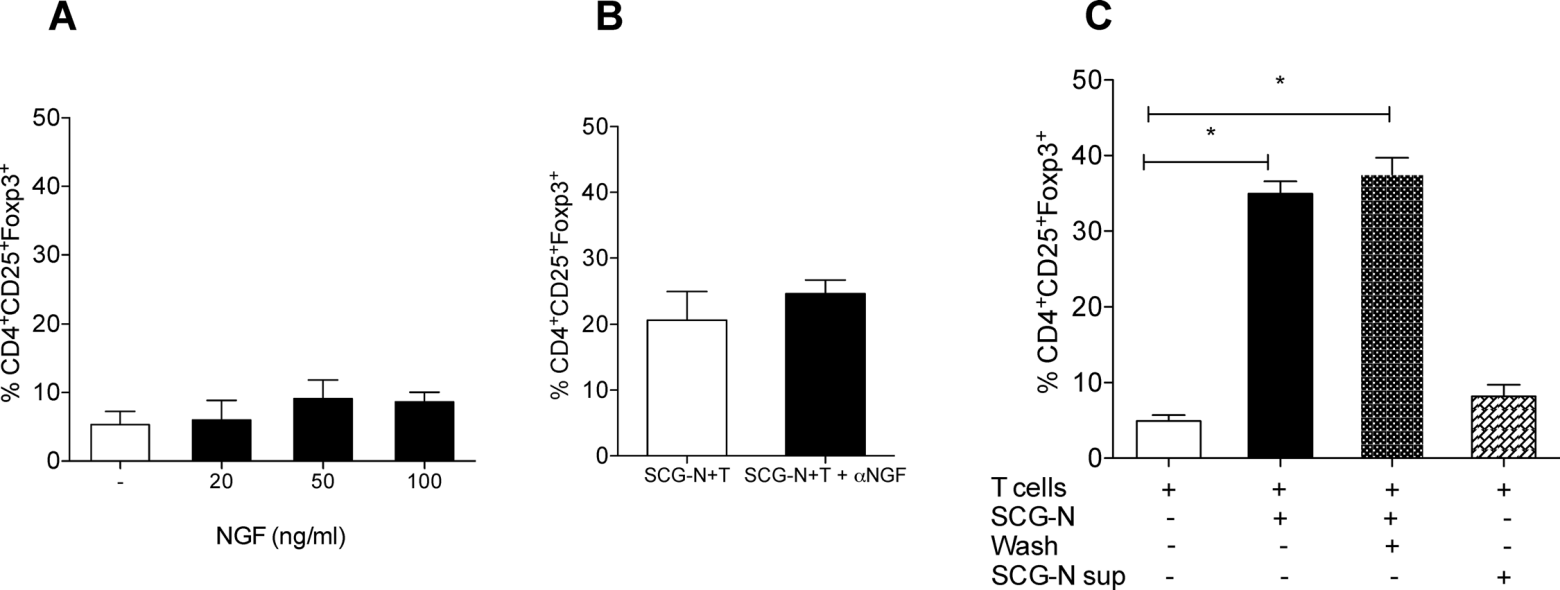
Neuronal generation of Tregs is independent of NGF. (A) Various concentrations of NGF were added to T cells and incubated for 5 days (n = 6). (B) Anti-NGF was added to the neuron culture one hour before adding T cells and left for 5 days in culture (n = 6). (C) Supernatant from neuronal culture was washed away before adding T cells to the neuron culture and added to CD4^+^ T cells (n = 6). T cells were stained with antibodies against CD3, CD4, CD25 and Foxp3; and analyzed by flow cytometry. Mean ± SEM percentage of Foxp3 expression among the CD3^+^CD4^+^CD25^+^ cells (* = p<0.0001).

Neuron growth needs NGF and our co-culture system was established by culturing SCG-neurons in the presence of 100ng/ml NGF for 2 days followed by adding naïve CD4^+^ T cells to the neuron cultures. To investigate the effect of NGF present in neuron culture on induced Foxp3 expression in T cells, we either added NGF to T cells in various concentrations (0, 20, 50 and 100 ng/ml) or neutralized NGF in the co-cultures with specific antibody. NGF at any concentration had no significant effect on the induction of Foxp3 expression in T cells (Fig 3A) and NGF neutralizing antibody resulted in no changes in induction of Foxp3^+^ cells in the co-culture (Fig 3B). Interestingly, the neurons survive without additional NGF where T cells are present in the co-cultures, while in the absence of the T cells or NGF the neurons did not survive (data not shown).

Additionally, the possible effect of NGF and other factors present in the supernatant of neuronal culture before the addition of T cells was examined by washing neurons before initiating the co-culture. The induced Foxp3 expression in CD4^+^CD25^+^ cells from this co-culture (37.50±2.20%) was compared with the Foxp3 expression in CD4^+^CD25^+^ cells from the co-culture with no removal of supernatant of the neurons (34.95±1.65%) (Fig 3C). In addition, the removed supernatant was added to the naïve T cells and no significant induction of Foxp3 expression was demonstrated (Fig 3C).

### Up-regulation of Foxp3 expression in the co-culture is accompanied by a reduction in the percentage of IFNγ producing cells

**Fig 4:**
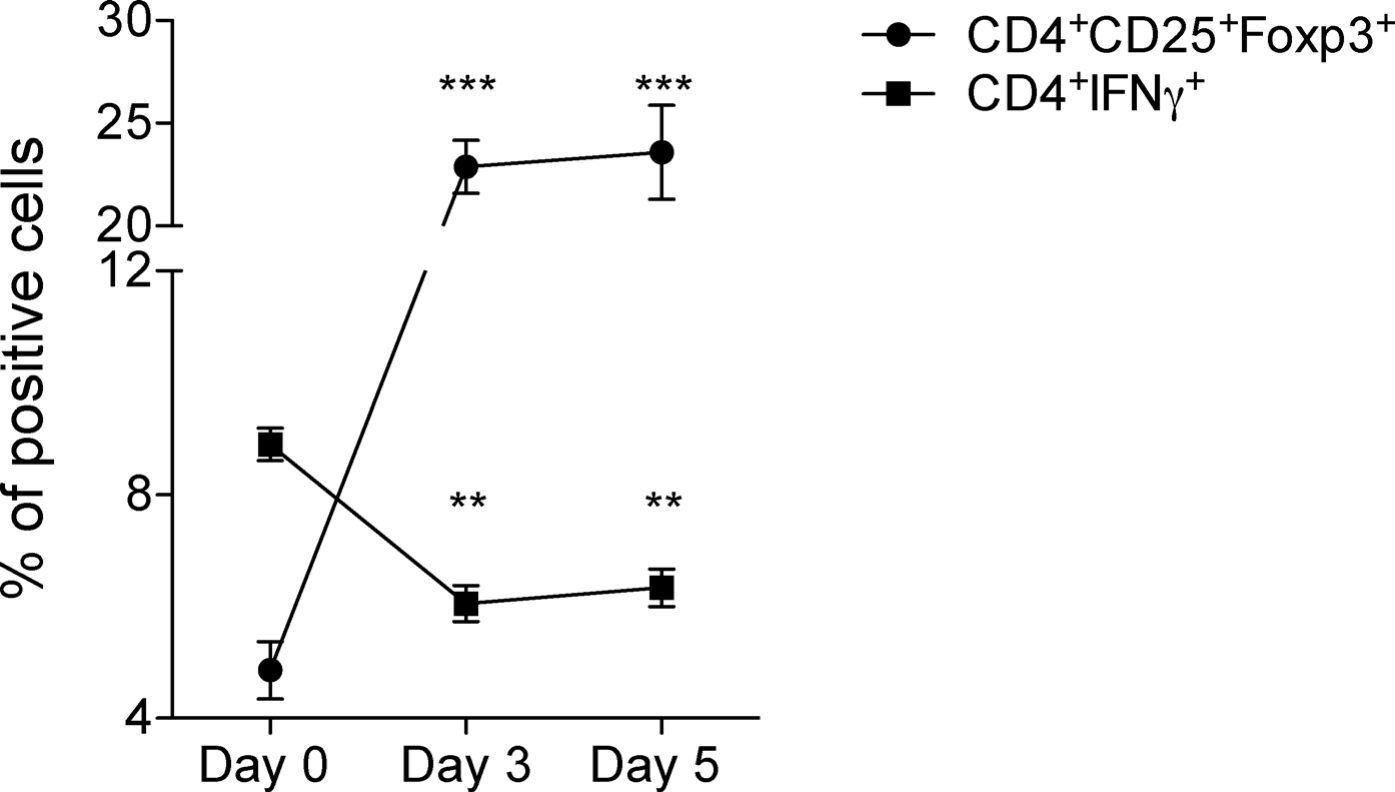
Up-regulation of Foxp3 expression in neuron-T cell co-culture is accompanied by a reduction in the levels of IFN-γ expression. CD4^+^ T cells were co-cultured with SCG-neurons and at indicated time stained with antibodies against CD3, CD4, CD25, Foxp3, and IFN-γ. The cells were analyzed by flow cytometry. Mean ± SEM percentage of the expression of Foxp3 among CD3^+^CD4^+^CD25^+^ cells and IFN-γ among CD3^+^CD4^+^ cells are depicted. (n = 4, ** = p<0.0002, *** = p<0.0001).

Induction of Tregs in neuron-T cell co-culture displayed the effect of neurons on T cells. Next, we questioned whether neurons and T cell co-cultures also influenced T cell functions. We measured the levels of cells that express the proinflammatory cytokine, IFNγ. IFNγ production was measured and observed over a time course period and determined by flow cytometry. A significant decrease in the level of IFNγ production was detected, whereas the expression of Foxp3 significantly increased (Fig 4). This may be due to the presence of a regulatory pathway in which the induction of Tregs down-regulates the production of pro-inflammatory cytokines, such as IFNγ.

### The neuropeptide CGRP but not vasoactive intestinal peptide (VIP) is needed for neuronal generation of Tregs

**Fig 5:**
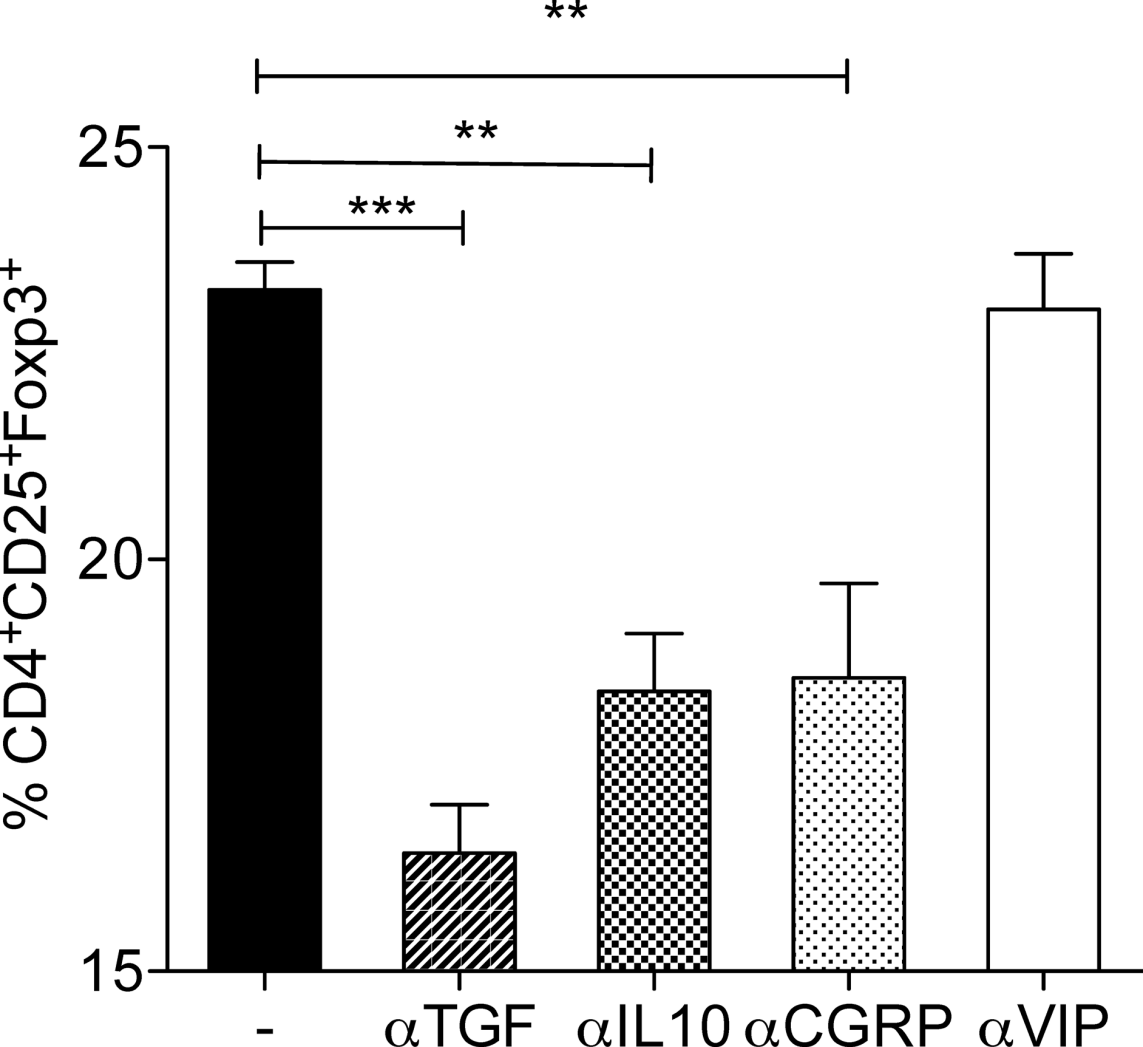
CGRP but not VIP contributes to the generation of Foxp3^+^ Tregs by SCG-neurons. Neuron cultures were incubated with neutralization antibodies for TGF-β, IL-10, or VIP, and CGRP antagonist for one hour before adding T cells. The co-cultures were incubated for 5 days and T cells were stained for CD3, CD4, CD25 and Foxp3 and were analyzed using flow cytometry. Mean ± SEM percentage of Foxp3 expression among CD3^+^CD4^+^CD25^+^ cells in different co-cultures are shown. (n = 6, ** = p<0.002, *** = p<0.0002).

**Fig 6:**
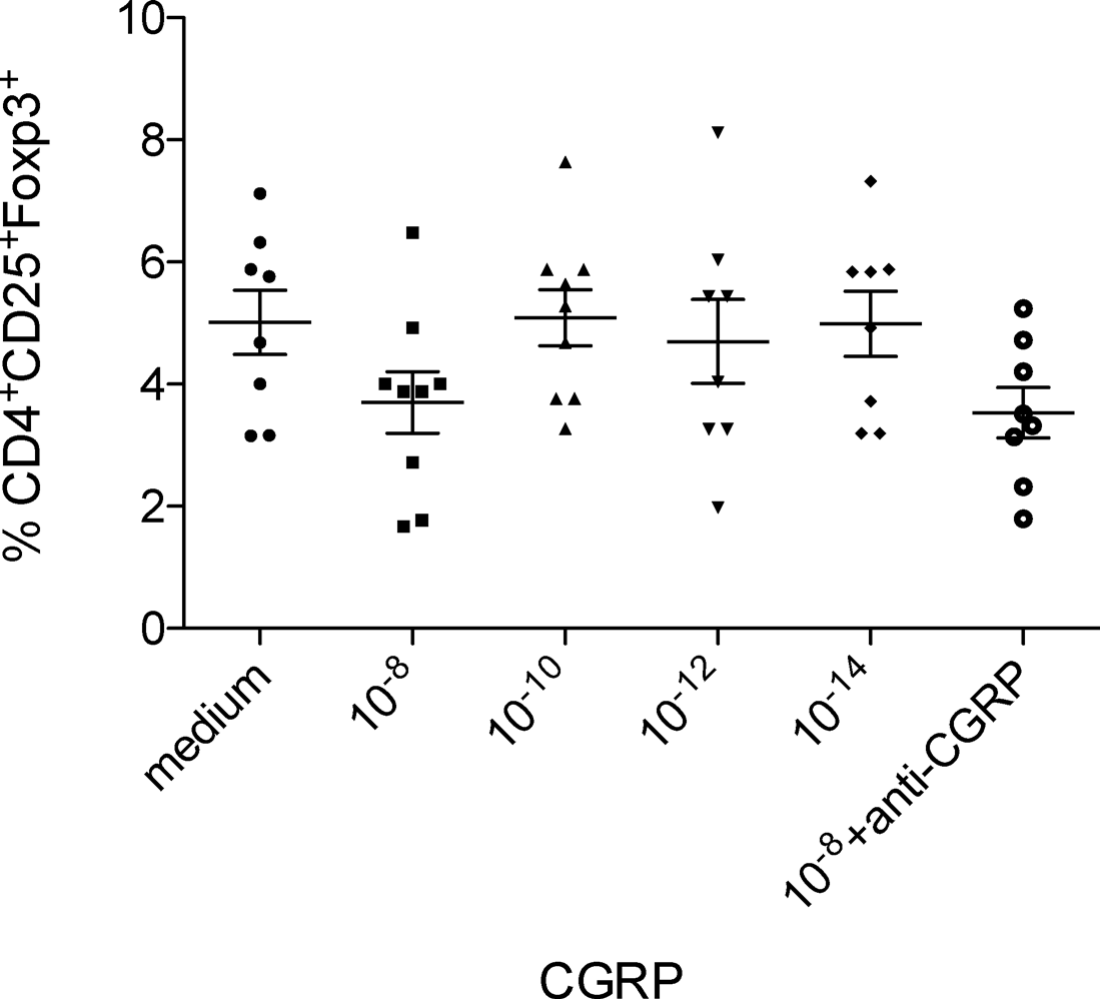
CGRP fails to induce Tregs in CD4^+^ T cell population. Various concentrations of CGRP were added to T cells and incubated for 3 days. Where indicated Anti-CGRP was added to the T cell culture for one hour and cells were washed before adding CGRP. T cells were stained with antibodies against CD3, CD4, CD25 and Foxp3; and analyzed by flow cytometry. Each dot represents the percentage of Foxp3 expression among the CD3^+^CD4^+^CD25^+^ cells at indicated concentration. Data are from two independent experiments.

Tregs may be induced by TGF-β[2], IL-10[24], and VIP [25]. Additionally, CGRP is shown to inhibit DC maturation and CGRP-pretreated DC induce increased numbers of Tregs [26]. Addition of anti-TGF-β, anti-IL-10, and CGRP antagonist to T cell-neuron co-culture significantly down-regulated the percentages of Foxp3^+^CD4^+^CD25^+^ T cells whereas anti-VIP had no significant effects compared to Foxp3 expression in T cells co-cultured with neurons (Fig 5). Moreover, culture of T cells (CD4+ or CD4+ CD25-) with different concentrations of CGRP (10–14 to 10–8) in the absence of neurons failed to induce any foxp3+ Tregs (Fig 6).

## Discussion

The current study shows that SCG-neurons can interact with naïve CD4^+^ T cells and that neurons induce Foxp3 expression in such cells through the immunoregulatory cytokines, TGF-β and IL-10, as well as the neuropeptide CGRP. Neuronal generation of Tregs involving TGF-β in our studies is consistent with Liu et al.’s [2] who showed in an animal model of experimental autoimmune encephalomyelitis (EAE) that the interaction between neurons and T cells resulted in the conversion of encephalitogenic T cells to Tregs. However, our studies were performed in a situation that reflects non-inflammatory situation in the body, using SCG neurons and T cells from naive BALB/c mice, indicating neuron-T cell interaction is an on-going cross-talk, which may constitutively affect immune homeostasis.

Our experiments show that Foxp3 up-regulation is not only induced in total splenocytes but also in purified CD4^+^ and naïve CD4^+^CD62L^+^ T cells after co-culture with neurons. This suggests that neuron-CD4^+^ T cell interactions alone are required for the expression of this phenomenon and that accessory cells in the spleen are not responsible for the induction of expressed Foxp3.

Treg induction in our co-culture system was partially dependent on TGF-β. TGF-β plays an essential role in differentiation of Foxp3^+^ Tregs. These Tregs secrete TGF-β, which acts in an autocrine manner and also plays a role in suppressing other immune cells. Furthermore, TGF-β converts CD4^+^CD25^-^ T cells into Foxp3 expressing CD4^+^CD25^+^ T cells, which in turn, produce TGF-β [17, 27–29]. The presence of IL-2 is necessary for this conversion to take place and TGF-β is essential for T cells to become Foxp3^+^ Tregs [29]. Liu et al. [2] reported that cerebellar neurons produce TGF-β, however, it is unknown if SCG neurons also produce TGF-β under co-culture conditions. More studies are required to clarify this issue further.

IL-10 is an immune regulatory cytokine produced by Tregs and other immune and non-immune cells such as gut epithelium [30–32]. TGF-β is essential for Tregs to produce IL-10 [29, 32]. We observed IL-10 to play a role in the induction of Foxp3 expression in T cells when co-cultured with neurons. IL-10 appears to be essential for maintaining Foxp3^+^ Treg differentiation in colitis [33]. This might be a positive feedback mechanism as IL-10 is secreted by Foxp3^+^ Tregs to maintain the Foxp3 expression. It is an option that the TGF-β present in the co-culture induced Foxp3^+^ Tregs that are responsible for production of IL-10, which in an autocrine manner and in co-operation with TGF-β maintains the Foxp3 expression.

IL-10 protects neurons through the IL-10 receptor, which is expressed on spinal cord neurons and cortical neurons [34, 35]. It is unknown if SCG-neurons express the IL-10 receptor, but IL-10 produced by Tregs in the co-culture is an indication of Treg involvement in the survival of neurons that in turn could further induce and maintain Foxp3 expression.

VIP is a neuropeptide with significant immunoregulatory effects. It is systemically distributed and produced by nerves and various kinds of immune cells; mainly T cells [36, 37]. VIP contributes to the generation of Foxp3 expressing IL-10 and TGF-β producing Tregs in severe inflammatory situation like arthritis [25, 38, 39]. It is possible however, that an inflammatory microenvironment is required for VIP-induced Tregs, which is absent in our co-culture system.

CGRP is a neurotransmitter secreted by neurons throughout the whole body. It is also a potent immunoregulatory and anti-inflammatory neuropeptide secreted under physiologic conditions and is capable of complete suppression of cutaneous hypersensitivity reactions [40]. It has been shown to inhibit macrophage antigen presentation, B cell antibody secretion and T cell functions [41–43]. Interestingly, macrophages, T cells and Langerhans cells express CGRP receptors and CGRP modulates the immune responses through receptor binding [44, 45]. Additionally, CGRP is shown to regulate cutaneous immunity under physiological conditions [45]. Current available evidence suggests that, in neuro-immunological interactions, CGRP mediates a potent peptidergic anti-inflammatory environment/modulation by distinct pathways involving the amplification of IL-10 production [46]. In addition, *in vitro* modulation of DC-induced T cell immune responses and increased numbers of CD4^+^CD25^+^Foxp3^+^ T cells by CGRP has been demonstrated [26]. To the best of our knowledge, our work is the first finding providing evidence that in a coculture system neuronal CGRP promotes Foxp3 expression in naïve T cells.

SCG neuron culture supernatant failed to induce FOXP3 expression in T cells, however, Treg induction in the co-culture system was mediated by secreted IL-10, TGF beta, and CGRP. These findings suggest the requirement of cell-to-cell contact as well as soluble factors produced in the cocultures for generation of Tregs by SCG neurons. These findings were supported by incubation of CD4+ T cells with different concentrations of CGRP in the absence of SCG neurons. CGRP failed to induce Foxp3 Tregs in purified CD4+ T cells at all concentrations examined (10^−14^ to 10^−18^, Fig 6)

We used NGF, a neuronal growth factor, to aid the growth and survival of cultured neurons in our study. NGF is synthesized by nerves but also by a variety of cell types such as immune cells like T- and B-lymphocytes, eosinophils and macrophages, as well as non- immune cells such as epithelial cells [47–51]. These immune cells express specific NGF receptors indicating that NGF interacts with and influences both the adaptive and innate immune systems [52]. However, our findings ruled out the requirement and involvement of NGF in generation of Tregs in neuron-T cell cultures. Neither neutralization of NGF with antibody or incubation of purified naïve T cells with NGF led to any change in the numbers of Foxp3 expressing cells in our co-cultures. Surprisingly, we found prolongation in the survival of neurons in our co-cultures in the absence of added NGF or its neutralization, suggesting that T cells clearly secrete a factor or factors to maintain the survival of co-cultured neurons, which by themselves will not survive in its absence [53, 54]. Overall this indicates the beneficial effects of neuron-T cell association and suggests that similar effects may occur in *vivo*. For instance, in vivo induction of tolerance could occur as a result of communication between T cells and other innate and adaptive immune components as reported for B cells [55–57] and hepatocytes[58, 59].

GM-CSF is produced by mouse SCG neurons[60] and demonstrated to stimulate sympathetic nerve growth[61]. Additionally, a dual role for GM-SCF[62] as a pro-inflammatory cytokine that stimulates immune response or a regulatory factor, which recruit Treg has been proposed. GM-CSF demonstrated to interact with CD116 on Treg to promote immune tolerance[63]. It is remain to be investigated whether GM-CSF is produced by SCG neurons in our coculture system to participate in the induction of Foxp3+ Treg cells.

The presence of IL-2 improves the viability of Tregs [29, 64]. IL-2 is not necessary for the thymic development of Tregs but is critical for the maintenance and survival of mature Tregs [65–67]. Although, in the absence of IL-2 the effect of neurons to generate Foxp3^+^ Tregs was demonstrable, addition of IL-2 promoted the Foxp3 expression in the co-cultures. This suggests that neuronal generation of Tregs is independent of IL-2.

In addition to modulated Foxp3 expression upon neuron and T cell co-culture, we also observed downregulation of IFNγ production by T cells. This reduced expression of IFNγ could be due to several factors including the presence of TGF-β [29, 64–66] and CGRP [25, 45] in the co-cultures. Interestingly, several studies indicate that various components of the immune system are involved in the process of neurogenesis [68]. Evidence for a role of T cells in neurogenesis was provided by the demonstration that microglia activated by a low level of T cell-derived IFN-γ, promoted neurogenesis *in vitro* [69]. Modulation of T cells in our co-culture system to produce low levels of IFN-γ may have been a contributory factor in the support of neuronal growth.

This study provides evidence for further understanding the mechanisms behind the interaction between SCG-neurons and T cells that lead to the generation of Tregs and down-regulation of the pro-inflammatory cytokine, IFNγ. In summary, the increased Foxp3 expression in T cells in co-culture with SCG neurons is bi-directional, partially dependent on the immuno-regulatory cytokines TGF-β and IL-10, but also the neuropeptide CGRP.

## Supporting Information

S1 Fig Co-cultured SCG-neurons increase the frequency of Foxp3+ T cells. purified CD4+ T cells were co-cultured with neurons and percentage of Foxp3 expression was detected by flow cytometry on day 3 of coculture. Foxp3 expression significantly increased in CD4+ T cells in the co-cultures compared to purified CD4+ T cells that were left in culture media without neurons. No increase of Foxp3 expression was demonstrated in CD4+ T cells cultured with adherent cells isolated from spleen of syngeneic mice or the murine intestinal epithelial cell line MODE-K.
(TIFF)
