# Supplementary figures and images for: Superior Cervical Ganglia Neurons Induce Foxp3+ Regulatory T Cells via Calcitonin Gene-Related Peptide
Source: PLoS One. 2016 Mar 29;11(3):e0152443. doi: 10.1371/journal.pone.0152443 (PMC4811438; doi:10.1371/journal.pone.0152443)

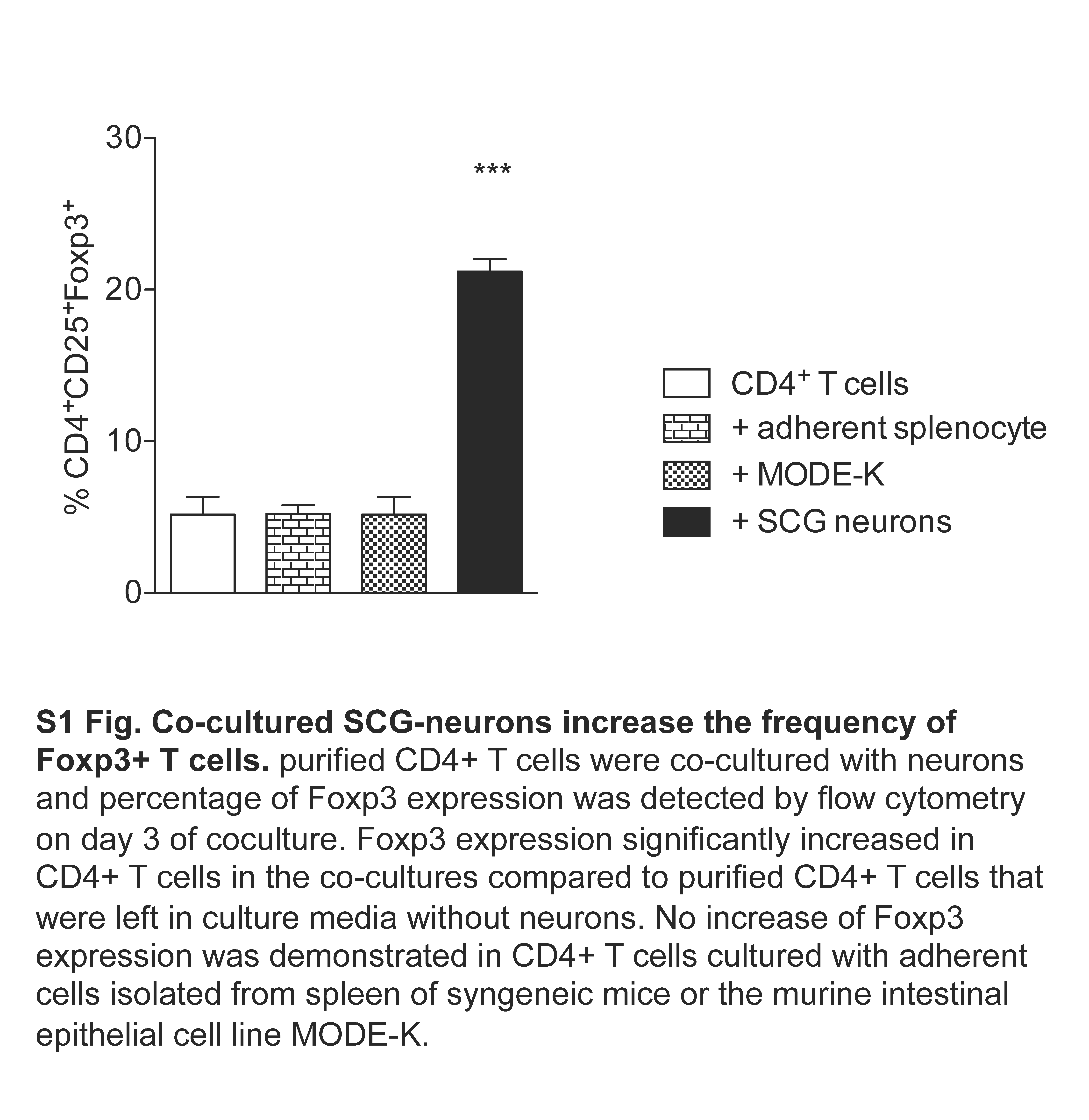

Supplement: S1 Fig — purified CD4+ T cells were co-cultured with neurons and percentage of Foxp3 expression was detected by flow cytometry on day 3 of coculture. Foxp3 expression significantly increased in CD4+ T cells in the co-cultures compared to purified CD4+ T cells that were left in culture media without neurons. No increase of Foxp3 expression was demonstrated in CD4+ T cells cultured with adherent cells isolated from spleen of syngeneic mice or the murine intestinal epithelial cell line MODE-K. (TIFF) [file pone.0152443.s001.tiff]
